# m5C and m6A cooperatively stabilize EPHB4 to drive lymphatic metastasis in gastric cancer

**DOI:** 10.3389/fgene.2026.1845032

**Published:** 2026-05-20

**Authors:** Yi Zhang, Ruofan He, Xinjian Lin, Jianxin Ye

**Affiliations:** 1 Department of Gastrointestinal Surgery 2 Section, the First Affiliated Hospital of Fujian Medical University, Fuzhou, China; 2 Key Laboratory of Gastrointestinal Cancer (Fujian Medical University), Ministry of Education, Fuzhou, China; 3 Fujian Key Laboratory of Tumor Microbiology, Department of Medical Microbiology, School of Basic Medical Sciences, Fujian Medical University, Fuzhou, China

**Keywords:** EphB4, gastric cancer, lymphatic metastasis, m5C modification, m6A modification

## Abstract

**Background:**

Gastric cancer remains a major cause of cancer-related mortality worldwide, and lymph node metastasis is a major determinant of recurrence and poor prognosis. However, the molecular basis of lymphatic dissemination, particularly the epitranscriptomic regulation of metastasis-related genes, remains unclear. We investigated whether RNA modifications promote gastric cancer lymphatic metastasis by regulating EPHB4, a receptor tyrosine kinase implicated in lymphangiogenesis and nodal spread.

**Methods:**

We integrated transcriptome sequencing of gastric cancer tissues, bioinformatic database analyses, and immunohistochemical validation to identify EPHB4 as a metastasis-associated gene. *In vitro* gain- and loss-of-function assays and an *in vivo* hindfoot lymphatic metastasis mouse model were used to assess the role of EPHB4 in metastatic behavior. Mechanistic studies, including RNA stability assays, RIP, MeRIP-qPCR, dual-luciferase reporter assays, RIPiT, co-immunoprecipitation, and confocal microscopy, were performed to determine how NSUN2/YBX1-mediated m^5^C regulation and IGF2BP1-associated m^6^A recognition affect EPHB4 mRNA.

**Results:**

Transcriptome sequencing identified EPHB4 as significantly upregulated in lymph node-positive gastric cancer. Database analyses and immunohistochemistry further supported the association of high EPHB4 expression with poor clinical outcome. Functionally, EPHB4 promoted lymphatic metastasis *in vivo* and enhanced migration and invasion of gastric cancer cells *in vitro*. Mechanistically, NSUN2, YBX1, and IGF2BP1 each increased EPHB4 expression by stabilizing its mRNA. NSUN2 deposited m^5^C marks on EPHB4 transcripts that were recognized by YBX1, whereas IGF2BP1 preferentially bound m^6^A-modified EPHB4 RNA and supported its stability. Mutational mapping and dual-luciferase assays identified functional m^5^C- and m^6^A-associated sites within EPHB4 mRNA. YBX1 and IGF2BP1 also physically interacted, co-occupied EPHB4 transcripts, and reciprocally enhanced m5C- and m6A-associated EPHB4 RNA enrichment.

**Conclusion:**

These findings reveal a cooperative epitranscriptomic mechanism in which NSUN2/YBX1-mediated m^5^C signaling and IGF2BP1-associated m^6^A recognition converge on EPHB4 mRNA to promote gastric cancer lymphatic metastasis, highlighting the NSUN2-YBX1-IGF2BP1-EPHB4 axis as a potential therapeutic target.

## 1 Introduction

Gastric cancer remains one of the most common malignant tumors worldwide and continues to impose a substantial global health burden (Bray et al., 2024). Although surgical techniques, perioperative management, chemotherapy, and molecularly targeted therapies have improved, many patients still present with advanced disease, and lymph node metastasis remains a major cause of recurrence and poor survival (Bruno et al., 2000; Chen et al., 2002). Because nodal dissemination strongly influences disease staging, postoperative management, and long-term outcome, defining the molecular mechanisms that drive lymphatic metastasis is of considerable biological and clinical importance.

EPHB4 (ephrin type-B receptor 4) is a member of the Eph receptor tyrosine kinase family and regulates a broad range of physiological and pathological processes (Arcas, Wilkinson, and Nieto, 2020). In particular, EPHB4 has a well-established role in vascular and lymphatic development (Zhang et al., 2015). In gastric cancer, elevated EPHB4 expression has been associated with deeper invasion, lymph node metastasis, and poor prognosis (Yin et al., 2017). Despite this evidence, the upstream mechanisms responsible for EPHB4 dysregulation in gastric cancer remain poorly defined, especially at the post-transcriptional level.

Among post-transcriptional regulatory mechanisms, RNA methylation has emerged as a major determinant of cancer cell behavior. N6-methyladenosine (m^6^A) and 5-methylcytosine (m^5^C) are two extensively studied RNA modifications that influence transcript stability, localization, and translation. NSUN2, a key m^5^C methyltransferase, is upregulated in multiple cancers and can promote malignant phenotypes by stabilizing selected transcripts (Mei et al., 2020). YBX1 and IGF2BPs are RNA-binding proteins that act as modification-sensitive effectors and control the fate of methylated RNAs(Xu et al., 2025; Xu et al., 2024; Lin et al., 2026). Both proteins are aberrantly expressed in gastric cancer and have been implicated in the stabilization of oncogenic transcripts (Qin et al., 2024; Luo and Lin, 2022). However, whether m^5^C- and m^6^A-related regulators cooperate to control EPHB4 expression during gastric cancer lymphatic metastasis has not been established.

Here, we show that EPHB4 is enriched in lymph node-positive gastric cancer and functionally promotes lymphatic dissemination. We further demonstrate that NSUN2-dependent m^5^C deposition, YBX1-mediated recognition of m5C-marked transcripts, and IGF2BP1-associated recognition of m^6^A-modified EPHB4 transcripts act in a coordinated manner to stabilize EPHB4 mRNA. These findings uncover an epitranscriptomic mechanism that links RNA modification crosstalk to EPHB4-driven lymphatic metastasis in gastric cancer.

## 2 Materials and methods

### 2.1 Cell lines and culture conditions

AGS and HGC27 gastric cancer cell lines, together with human lymphatic endothelial cells (HLECs), were purchased from the Shanghai Institutes of Biological Sciences, Chinese Academy of Sciences (Shanghai, China). HEK293T cells were obtained from the American Type Culture Collection (ATCC, Manassas, VA, United States). All cell lines were maintained in the recommended culture media supplemented with 10% fetal bovine serum (FBS; PAN Seratech, Eidenbach, Germany) at 37 °C in a humidified incubator containing 5% CO_2_.

### 2.2 Generation of stable shRNA knockdown cell lines

Short hairpin RNAs (shRNAs) targeting EPHB4 and a non-targeting negative control were synthesized and cloned into the AgeI and EcoRI sites of pLKO.1-puro (8453; Addgene, Watertown, MA, United States). Lentiviral particles were generated by co-transfecting the shRNA plasmids with the packaging vectors p-VSVG and p-PHR into HEK293T cells. Viral supernatants were used to infect HGC27 cells, and infected cells were selected with puromycin (2 ug/mL) for 72 h beginning 48 h after infection. Knockdown efficiency was verified by Western blotting. shRNA sequences are listed in Supplementary Table S1.

### 2.3 Immunohistochemistry (IHC)

Freshly isolated tissues were fixed in 4% paraformaldehyde overnight at room temperature, embedded in paraffin, sectioned, deparaffinized, and rehydrated. Heat-induced antigen retrieval was performed in sodium citrate buffer. After blocking for 1 h at room temperature, sections were incubated with primary antibodies overnight at 4 °C, followed by incubation with secondary antibodies (PV-9001/2; ZSGB-BIO, Beijing, China). Staining was visualized using a DAB substrate kit (ZLI-9018; ZSGB-BIO). Staining intensity was scored on a four-tier scale in 200× fields as follows: 0, no staining; 1, weak staining (light yellow); 2, moderate staining (yellow-brown); and 3, strong staining (brown).

### 2.4 Hindfoot lymphatic metastasis model

All animal experiments were approved by the Institutional Animal Care and Use Committee of Fujian Medical University (Approval No. IACUC FJMU 2023-Y-1139) and were performed in accordance with the National Institutes of Health Guide for the Care and Use of Laboratory Animals and the ARRIVE guidelines. Male nude mice (4–5 weeks old) were injected in the footpad with 1 x 10^6^ cells suspended in 20 uL PBS. At the experimental endpoint, mice were euthanized by gradual CO_2_ displacement, followed by cervical dislocation as a secondary confirmation of death. Regional draining lymph nodes were harvested for subsequent analysis. Metastatic human tumor cells within lymph nodes were detected using an anti-human mitochondria antibody, and the lymph node metastasis rate was calculated accordingly.

### 2.5 RNA interference

Small interfering RNAs (siRNAs) targeting EPHB4, NSUN2, YBX1, and IGF2BP1 were synthesized by GenePharma (Shanghai, China). AGS and HGC27 cells were transfected using JetPrime (Polyplus, Strasbourg, France) according to the manufacturer’s protocol. A scrambled siRNA served as the negative control. siRNA sequences are listed in Supplementary Table S1.

### 2.6 Cell-surface crosslinking and pull-down assay

HLECs were grown to approximately 80% confluence and labeled with Sulfo-NHS-SS-Biotin dissolved in ice-cold PBS to a final concentration of 0.5 mg/mL, followed by incubation on ice for 30 min. The reaction was quenched with 100 mM glycine in ice-cold PBS for 15 min. Biotinylated HLECs and HGC27 cells were then detached, mixed at a 1:1 ratio in pre-warmed PBS containing calcium and magnesium, and co-incubated at 37 °C for 60 min. Freshly prepared BS3 crosslinker was added to a final concentration of 1–2 mM and incubated on ice for 30 min. After quenching with 100 mM Tris-HCl (pH 7.5), cells were lysed in western/IP lysis buffer, incubated with Strep-Tactin magnetic beads overnight, and analyzed by Western blotting.

### 2.7 Western blotting

Cells were lysed in RIPA buffer supplemented with protease inhibitors and PMSF. Protein concentrations were measured using a BCA assay. Equal amounts of protein (50 ug) were separated by SDS-PAGE, transferred to PVDF membranes, and blocked with 5% bovine serum albumin. Membranes were incubated with primary antibodies overnight at 4 °C and then with HRP-conjugated secondary antibodies for 1 h at room temperature. Protein bands were visualized using an enhanced chemiluminescence system. Primary antibodies included anti-NSUN2, anti-YBX1, anti-IGF2BP1, anti-GAPDH, and anti-EPHB4. Original blot images used for analysis are provided in the Supplementary Material.

### 2.8 Wound-healing assay

Cells (5 x 10^5^ per well) were seeded into six-well plates. After the cells reached near confluence, a sterile 200-uL pipette tip was used to generate a scratch across the monolayer. Detached cells were removed by washing with PBS, and the cultures were then maintained in serum-free medium. Images were captured at 0 and 48 h, and wound closure was quantified using ImageJ.

### 2.9 Transwell invasion assay

Cell invasion was assessed using 24-well Transwell inserts (8-um pore size) precoated with Matrigel. Cells (5 x 10^5^) were resuspended in 200 uL serum-free medium and seeded into the upper chamber. The lower chamber contained 600 uL complete medium supplemented with 10% FBS as a chemoattractant. After 48 h, non-invading cells were removed from the upper surface of the membrane. Cells that had invaded to the lower surface were fixed with 4% paraformaldehyde, stained with 0.1% crystal violet, and counted microscopically.

### 2.10 RNA extraction and RT-qPCR

Total RNA was extracted from gastric cancer cells using TRIzol reagent according to the manufacturer’s protocol. Complementary DNA was synthesized and subjected to quantitative PCR using the SYBR Green Pro Taq HS qPCR Kit (Accurate Biology, Changsha, China). GAPDH served as the internal control. Primer sequences are listed in Supplementary Table S2.

### 2.11 RNA stability assay

AGS and HGC27 cells were treated with actinomycin D (5 ug/mL) for 0, 2, 4, and 6 h. Total RNA was then isolated, and EPHB4 mRNA abundance was measured by RT-qPCR. The degradation constant (k) was calculated using the equation Nt/N0 = e^-kt^, where N0 is the RNA abundance at time 0 and Nt is the abundance at time t. The half-life (t1/2) was calculated as ln2/k.

### 2.12 RIP-qPCR

RIP assays were performed using the RNA Immunoprecipitation Kit (Geneseed, Guangzhou, China) according to the manufacturer’s instructions. Antibodies against NSUN2, YBX1, and IGF2BP1 were used for immunoprecipitation, and rabbit IgG served as the negative control. Co-precipitated RNA was purified and analyzed by RT-qPCR.

### 2.13 MeRIP-qPCR

Total RNA was extracted using TRIzol reagent. m^6^A or m^5^C RNA immunoprecipitation was then performed using the GenSeq m^6^A MeRIP Kit or the GenSeq m^5^C MeRIP Kit (CloudSeq Biotech, Shanghai, China) according to the manufacturer’s protocol. Enriched RNA was analyzed by RT-qPCR.

### 2.14 RNA immunoprecipitation in tandem (RIPiT)

HGC27 cells were lysed on ice in western/IP lysis buffer containing protease inhibitor cocktail and RNase inhibitor. Lysates were first incubated with Protein A/G magnetic beads and the indicated primary antibody for 12 h. After washing, immune complexes were eluted with 0.1 M glycine-HCl (pH 2.5–3.0), immediately neutralized with Tris-HCl (pH 8.0), and subjected to a second round of immunoprecipitation with the indicated antibody. RNA recovered from the final complexes was purified with TRIzol reagent and analyzed by RT-qPCR.

### 2.15 Luciferase reporter assay

Wild-type or mutant EPHB4 5′UTR, CDS, or 3′UTR fragments were cloned into the pmirGLO luciferase reporter vector. Cells were co-transfected with the reporter plasmids and the indicated siRNAs. After 48 h, luciferase activity was measured using the Dual-Glo Luciferase Assay System (Vazyme, Nanjing, China) according to the manufacturer’s instructions.

### 2.16 Co-immunoprecipitation (Co-IP)

Cells were lysed in western/IP lysis buffer supplemented with protease inhibitor cocktail and PMSF. Clarified lysates were incubated with Protein A/G magnetic beads and the indicated antibodies. Immunoprecipitated complexes were eluted and analyzed by Western blotting.

### 2.17 Confocal microscopy

AGS cells were fixed with 4% paraformaldehyde, permeabilized with 0.2% Triton X-100, and incubated with primary antibodies against FLAG, IGF2BP1, or YBX1. Appropriate Alexa Fluor 488- or Alexa Fluor 594-conjugated secondary antibodies were used for detection, and nuclei were counterstained with DAPI. Images were acquired using a Zeiss LSM-410 confocal microscope.

### 2.18 Clinical samples

A total of 22 gastric cancer tissue specimens were obtained from patients who underwent surgical resection at the First Affiliated Hospital of Fujian Medical University. All patients were pathologically diagnosed with primary gastric adenocarcinoma. Written informed consent was obtained from each participant, and the study was approved by the institutional ethics committee of Fujian Medical University (approval number: 202–166), in accordance with the Declaration of Helsinki. The cohort consisted of 22 male patients with gastric cancer, including 11 cases with positive lymph node metastasis and 11 cases without lymph node metastasis. Patient age ranged from 49 to 87 years, with a median age of 67 years. Based on the 8th edition of the AJCC staging system, 9 cases were classified as stage IIA, 2 as stage IIB, 6 as stage IIIB, and 5 as stage IIIC. All tumors were classified as the intestinal type according to the Lauren classification. Tumor tissues were used for EPHB4 immunohistochemical analysis.

### 2.19 Statistical analysis

Statistical analyses were performed using SPSS version 22.0, and graphs were generated with GraphPad Prism version 7. Data are presented as mean ± SD. Comparisons between two groups were performed using unpaired Student’s *t*-tests. Comparisons among multiple groups were performed using one-way ANOVA followed by LSD *post hoc* testing. A two-sided *P* value <0.05 was considered statistically significant.

### 2.20 RNA-seq and bioinformatics analysis

RNA sequencing was performed on eight gastric cancer tissue samples, including four tumors with lymph node metastasis and four without lymph node metastasis (Genome Sequence Archive for Human(Zhang S. et al., 2025): HRA014173). Library construction and paired-end sequencing was carried out by BGI Genomics Co., Ltd. on the DNBSEQ platform. Raw FASTQ reads were first subjected to quality control and adapter trimming using Trim Galore (Babraham Bioinformatics, Cambridge, United Kingdom; version 0.6.10). Clean reads were then aligned to the human reference genome (GRCh37/hg19) using HISAT 2 (version v2.2.2) (Kim et al., 2019). Transcript assembly and abundance estimation were performed using StringTie (version 1.3.6) in reference-guided mode with the corresponding gene annotation file using StringTie (Pertea et al., 2015), all default parameters were used in the analysis. Gene-level expression data were analyzed using DESeq2 (version 1.44.0) for normalization and differential expression analysis according to the standard DESeq2 workflow; genes with an adjusted *P* value <0.05 were considered significantly differentially expressed (Love, Huber, and Anders, 2014). Information on RNA-binding proteins and candidate RNA modification sites was retrieved from ENCORI, m5C-Atlas(Ma et al., 2022), and m6A-Atlas, restricting the analysis to human transcripts and the EPHB4 locus where applicable (Liang et al., 2024). TCGA stomach adenocarcinoma (TCGA-STAD) data were used to assess EPHB4 expression in normal and gastric cancer tissues (Cancer Genome Atlas Research Network, 2014), and survival analysis was performed using the Kaplan-Meier Plotter platform with default settings unless otherwise specified (Győrffy et al., 2013).

## 3 Results

### 3.1 EPHB4 is associated with lymphatic metastasis and promotes metastatic behavior in gastric cancer

**FIGURE 1:**
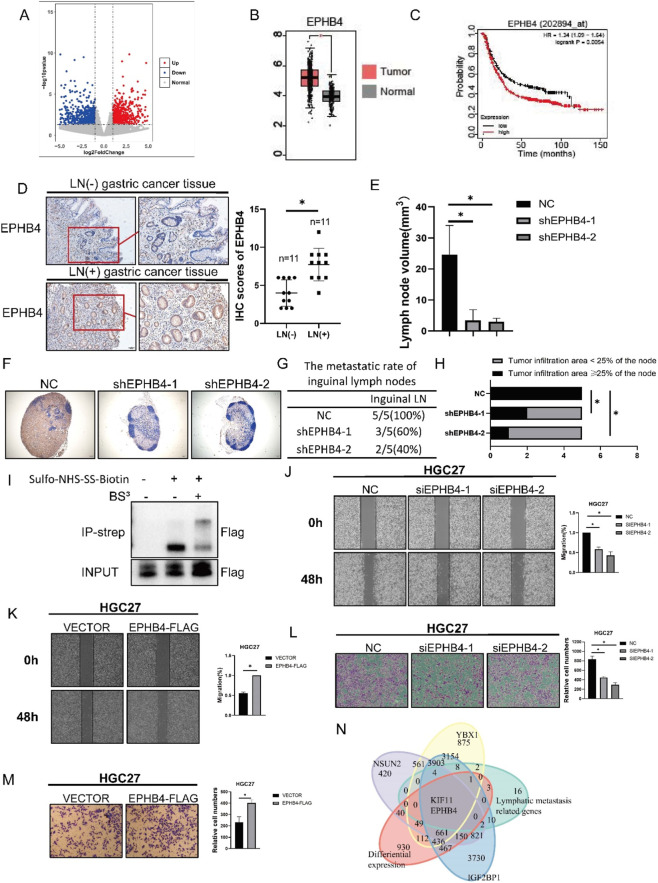
EPHB4 Promotes Lymphatic Metastasis in Gastric Cancer **(A)** Volcano plot of RNA sequencing of gastric cancer tissue samples lymph node metastasis or without lymph node metastasis. **(B)** Expression levels of EPHB4 in gastric cancer were analyzed using TCGA databases. **(C)** Prognostic significance of EPHB4 in gastric cancer were analyzed using KMplotter databases. **(D)** IHC staining of EPHB4 in gastric cancer tissues (n = 22). **(E)** The volume of inguinal lymph nodes (LNs) derived from HGC27 cells transfected with the pSilence vector in the hindfoot lymphatic drainage model. **(F)** IHC images of metastatic inguinal LNs, showing human mitochondria-positive HGC27 cells detected using an anti-human mitochondria antibody. Scale bars: 200 μm. **(G)** Metastasis ratios in dissected inguinal LNs. **(H)** Tumor infiltration in ≥25% of LNs was used to evaluate metastasis. Fisher’s exact test was applied. **(I)** Western blot of the complex formed between EPHB4-FLAG-tagged gastric cancer cells and biotin-labeled lymphatic endothelial cells. **(J)** Wound healing assay in HGC27 with EPHB4 knockdown. **(K)** Wound healing assay in HGC27 with EPHB4 overexpression. **(L)** Transwell invasion assay in HGC27 with EPHB4 knockdown. **(M)** Transwell invasion assay in HGC27 with EPHB4 overexpression. **(N)** Integration of differential expression analysis and CLIP-seq data identified EPHB4 as a common substrate of IGF2BP1, NSUN2 and YBX1. *p < 0.05.

To identify genes associated with lymphatic metastasis in gastric cancer, we performed RNA sequencing on advanced gastric cancer tissues with positive (N3a/N3b, M0) or negative (N0, M0) lymph node metastasis. Compared with lymph node-negative tumors, 803 genes were upregulated and 1,018 genes were downregulated in lymph node-positive specimens (Figure 1A). Integration of the differentially expressed genes with lymphangiogenesis-related gene sets identified KIF11 and EPHB4 as candidate genes linked to lymphatic metastasis. As the role of KIF11 in gastric cancer lymphatic metastasis has been investigated in a separate study,(Zhang et al., 2025a). We concentrated here on the second-ranked candidate, EPHB4, given its established function as a membrane receptor regulating lymphangiogenesis and its potential as a therapeutic target (Supplementary Table S3). Analysis of TCGA and KMplotter datasets further showed that EPHB4 was upregulated in gastric cancer and that higher EPHB4 expression was associated with worse prognosis (Figures 1B,C). Consistent with these findings, immunohistochemical analysis of gastric cancer tissues revealed significantly stronger EPHB4 staining in tumors with lymph node metastasis than in those without metastasis (Figure 1D).

To determine whether EPHB4 directly contributes to lymphatic dissemination, we generated stable EPHB4-knockdown gastric cancer cells and established a hindfoot lymphatic metastasis model. Silencing EPHB4 markedly reduced the size of regional draining lymph nodes and decreased the frequency of lymph node metastasis (Figures 1E–G). Immunostaining for human mitochondria further demonstrated a reduction in metastatic tumor cells within lymph nodes after EPHB4 knockdown (Figures 1F,H). Because tumor-cell EPHB4 can interact with ephrin-B2 on lymphatic endothelial cells (Zhang et al., 2015), we next performed BS3 crosslinking followed by Strep bead pull-down in a co-culture system. Overexpressed EPHB4-FLAG in HGC27 cells formed complexes with biotin-labeled surface molecules from lymphatic endothelial cells (Figure 1I), supporting direct intercellular contact. In parallel, wound-healing and Transwell assays showed that EPHB4 knockdown suppressed, whereas EPHB4 overexpression enhanced, the migratory and invasive capacities of gastric cancer cells (Figures 1J–M). Together, these data indicate that EPHB4 promotes lymphatic metastasis and pro-metastatic cell behavior in gastric cancer.

### 3.2 NSUN2, YBX1, and IGF2BP1 stabilize *EPHB4* mRNA

**FIGURE 2:**
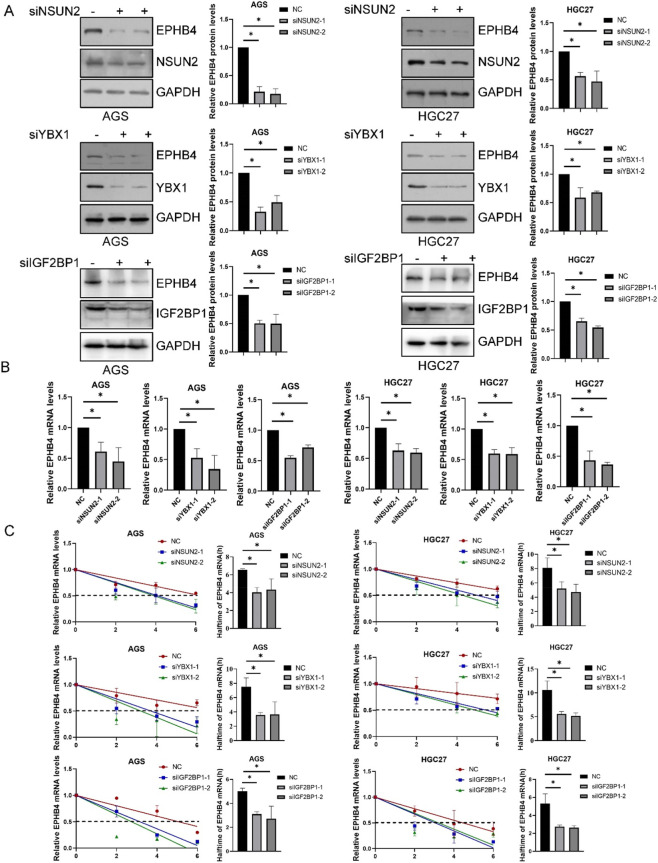
NSUN2, YBX1, and IGF2BP1 Mediate Post-Transcriptional Modification of EPHB4 **(A)** Western blot of EPHB4 in AGS and HGC27 cells following NSUN2, YBX1 or IGF2BP1 knockdown. **(B)** RT-qPCR of EPHB4 in AGS and HGC27 cells following NSUN2, YBX1 or IGF2BP1 knockdown. **(C)** Measurement of EPHB4 mRNA half-life. Data are means ± SD from three independent experiments. *p < 0.05.

Because EPHB4 expression appeared to be tightly controlled at the post-transcriptional level, we examined whether RNA modification regulators contribute to its expression. Transcriptome data showed that NSUN2, YBX1, and IGF2BP1 were all upregulated in lymph node-positive gastric cancer. StarBase analysis further predicted *EPHB4* mRNA as a common target of these three factors (Figure 1N), suggesting that EPHB4 may be jointly regulated by m^5^C- and m^6^A-related machinery. To test this hypothesis, we individually depleted NSUN2, YBX1, or IGF2BP1 in AGS and HGC27 cells. Knockdown of any of the three factors reduced both EPHB4 protein and mRNA levels (Figures 2A,B). Actinomycin D chase assays further showed that depletion of NSUN2, YBX1, or IGF2BP1 significantly shortened the half-life of *EPHB4* mRNA (Figure 2C). These findings indicate that NSUN2, YBX1, and IGF2BP1 each contribute to the maintenance of EPHB4 transcript stability.

### 3.3 NSUN2 installs m5C marks on EPHB4 mRNA and YBX1 recognizes the modified transcript

**FIGURE 3:**
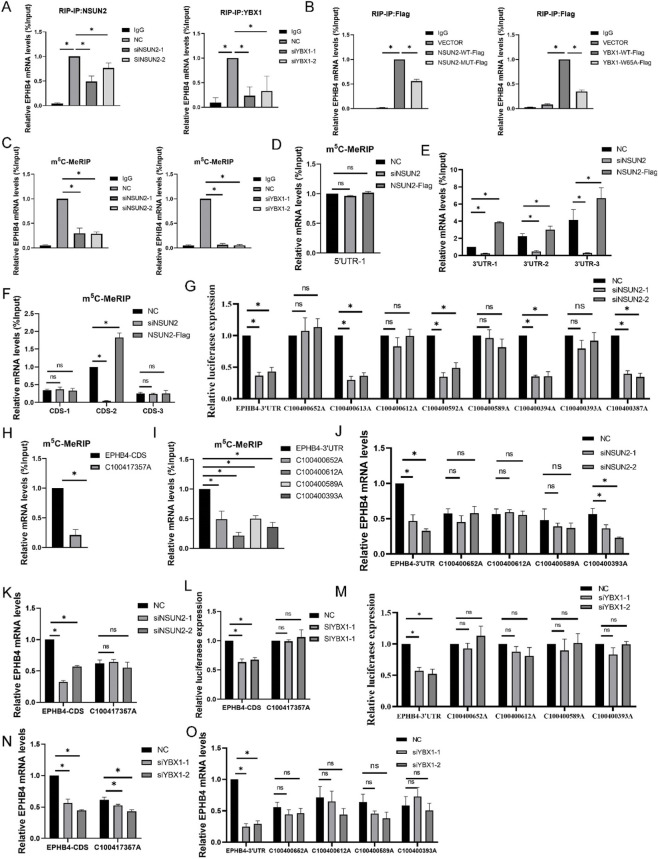
NSUN2 and YBX1 Mediate m^5^C Modification of EPHB4 mRNA **(A)** RIP-qPCR assay confirming the direct binding of NSUN2 and YBX1 to EPHB4 mRNA. **(B)** RIP-qPCR assay confirming the direct binding of NSUN2, YBX1, and their respective enzyme-inactive mutants to EPHB4 mRNA. **(C)** m^5^C-MeRIP-qPCR confirming m^5^C modification on EPHB4 mRNA. **(D)** m^5^C-MeRIP-qPCR confirming m^5^C modification on EPHB4 5′UTR mRNA. **(E)** m^5^C-MeRIP-qPCR confirming m^5^C modification on EPHB4 3′UTR mRNA. **(F)** m^5^C-MeRIP-qPCR confirming m^5^C modification on EPHB4 CDS mRNA. **(G)** Luciferase reporter assay showing relative luciferase activity (RLU) of wild-type or mutant EPHB4 3′UTR after NSUN2 knockdown. **(H)** m^5^C-MeRIP-qPCR confirming m^5^C modification on twild-type or mutant EPHB4 CDS. **(I)** m^5^C-MeRIP-qPCR confirming m^5^C modification on twild-type or mutant EPHB4 3′UTR. **(J)** RT-qPCR to detect the mRNA expression of EPHB4 3′UTR, C100417357A, C100400612A, C100400589A, and C100400393A after NSUN2 knockdown. **(K)** RT-qPCR to detect the mRNA expression of EPHB4 CDS and C100417357A after NSUN2 knockdown. **(L)** Luciferase reporter assay showing RLU of EPHB4 CDS and C100417357A after YBX1 knockdown. **(M)** Luciferase reporter assay showing RLU of EPHB4 3′UTR, C100417357A, C100400612A, C100400589A, and C100400393A after YBX1 knockdown. **(N)** RT-qPCR to detect the mRNA expression of EPHB4 CDS and C100417357A after YBX1 knockdown. **(O)** RT-qPCR to detect the mRNA expression of EPHB4 3′UTR, C100417357A, C100400612A, C100400589A, and C100400393A after YBX1 knockdown. *p < 0.05.

We next investigated whether NSUN2 and YBX1 regulate EPHB4 through an m^5^C-dependent mechanism. RIP assays showed that both NSUN2 and YBX1 associated with *EPHB4* mRNA, and this interaction was reduced after depletion of the corresponding protein (Figure 3A). Overexpression of wild-type NSUN2 or YBX1 increased their binding to *EPHB4* mRNA, whereas NSUN2 catalytically inactive mutant or YBX1 m5C-binding-deficient mutant did not produce the same effect (Figure 3B). Moreover, m^5^C-MeRIP-qPCR confirmed the presence of m5C modification on *EPHB4* mRNA, and the abundance of m^5^C-enriched EPHB4 transcripts decreased after NSUN2 or YBX1 knockdown (Figure 3C). These results support a model in which NSUN2-dependent m^5^C deposition and YBX1-associated recognition cooperate to stabilize *EPHB4* mRNA.

To localize the relevant m^5^C-responsive regions, we interrogated the m^5^C-Atlas database and found candidate m5C sites within the 5′UTR, coding sequence (CDS), and 3′UTR of EPHB4 (Supplementary Table S4). Fragment-based m^5^C-MeRIP-qPCR showed that NSUN2 knockdown or overexpression had little effect on the candidate 5′UTR region, whereas multiple 3′UTR fragments and one CDS-associated region displayed NSUN2-dependent changes in m^5^C enrichment (Figures 3D–F). We therefore generated luciferase reporters carrying wild-type or site-mutated EPHB4 fragments. Mutation analysis showed that loss of specific cytosines abolished or markedly attenuated the response to NSUN2 depletion, identifying C100417357, C100400652, C100400612, C100400589, and C100400393 as functionally important sites (Figures 3G–K). Consistent with a reader role for YBX1, depletion of YBX1 reduced the expression of reporters containing the intact NSUN2-responsive elements but did not further suppress constructs in which the critical m^5^C-associated residues had already been mutated (Figures 3L–O). Collectively, these results indicate that NSUN2 installs m5C marks on EPHB4 mRNA and that YBX1 recognizes these marks to promote transcript stability.

### 3.4 IGF2BP1 recognizes m^6^A-associated EPHB4 transcripts

**FIGURE 4:**
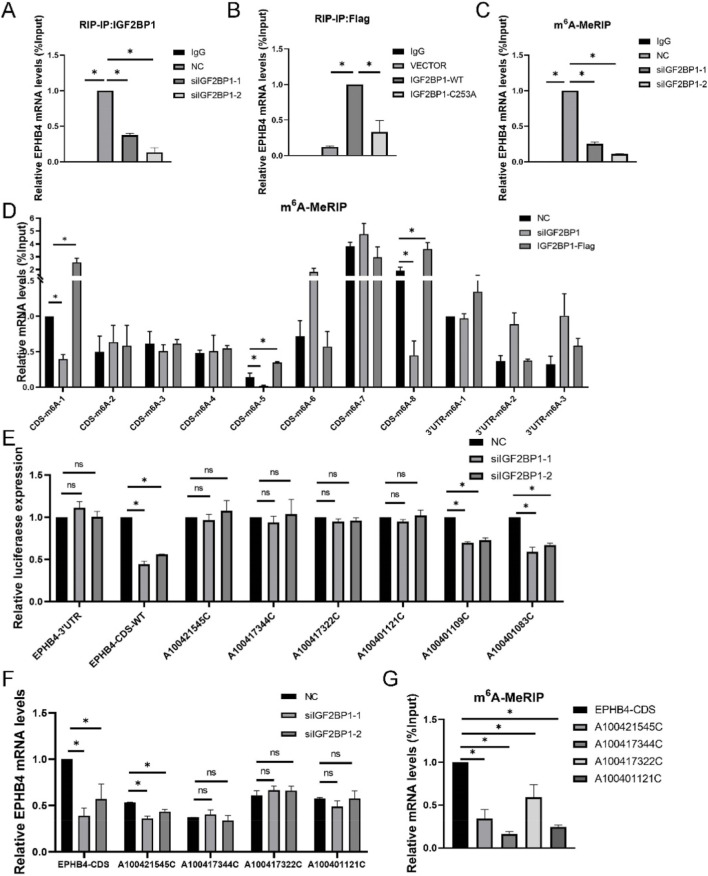
IGF2BP1 Mediates m^6^A Modification of EPHB4 mRNA **(A)** RIP-qPCR assay confirming the direct binding of IGF2BP1 to EPHB4 mRNA. **(B)** RIP-qPCR assay confirming the direct binding of IGF2BP1 and enzyme-inactive mutants to EPHB4 mRNA. **(C)** m^6^A-MeRIP-qPCR confirming m^6^A modification on EPHB4 mRNA. **(D)** m^6^A-MeRIP-qPCR confirming m^6^A modification EPHB4 CDS and 3′UTR mRNA. **(E)** Luciferase reporter assay showing RLU of EPHB4 3′UTR, EPHB4 CDS and the mutant of EPHB4 CDS after IGF2BP1 knockdown. **(F)** RT-qPCR to detect the mRNA expression EPHB4 CDS, A100421545C, A100417344C, A100417322C and A100401121C after IGF2BP1 knockdown. **(G)** m^6^A-MeRIP-qPCR confirming m^6^A modification on EPHB4 CDS, A100421545C, A100417344C, A100417322C and A100401121C. *p < 0.05.

We then examined whether IGF2BP1 regulates EPHB4 through an m^6^A-associated mechanism. RIP assays showed that EPHB4 mRNA was enriched in IGF2BP1 immunoprecipitates, and this enrichment decreased after IGF2BP1 knockdown (Figure 4A). Conversely, overexpression of wild-type IGF2BP1 increased its association with *EPHB4* mRNA, whereas mutation of the m^6^A-binding region abolished this effect (Figure 4B). m^6^A-MeRIP-qPCR demonstrated that EPHB4 transcripts carried detectable m^6^A marks and that the abundance of m^6^A-enriched EPHB4 RNA was reduced after IGF2BP1 depletion (Figure 4C), consistent with preferential binding of IGF2BP1 to m^6^A-modified EPHB4 transcripts.

Interrogation of m6A-Atlas identified candidate m6A sites in the CDS and 3′UTR of EPHB4 (Supplementary Table S5). Fragment-based m6A-MeRIP-qPCR revealed three responsive regions, namely, CDS-m6A-1 (A100421545), CDS-m6A-5 (A100417344 and A100417322), and CDS-m^6^A-8 (A100401121, A100401109, A100401083, A100401057, and A100401023), all of which showed decreased enrichment after IGF2BP1 knockdown and increased enrichment after IGF2BP1 overexpression (Figure 4D). To pinpoint the functional residues, we generated a series of site-mutated reporter constructs. Mutation analyses indicated that A100421545, A100417344, A100417322, and A100401121 were required for the IGF2BP1-dependent response, whereas the tested 3′UTR candidates did not show evidence of regulation by IGF2BP1 (Figures 4E–G). Together, these data indicate that IGF2BP1 recognizes m^6^A-associated EPHB4 transcripts and supports their stability through discrete regulatory sites.

### 3.5 YBX1 and IGF2BP1 cooperate to maintain EPHB4 expression

**FIGURE 5:**
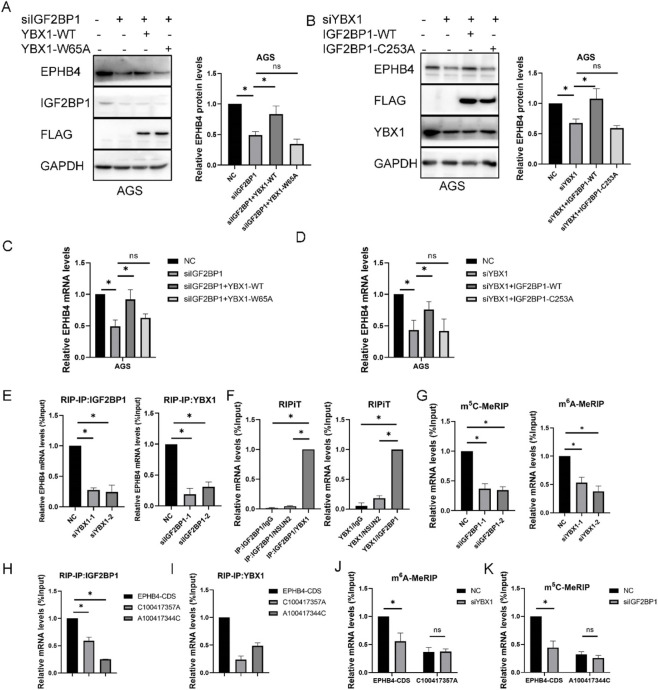
Cooperative Regulation of EPHB4 by YBX1 and IGF2BP1 **(A)** Western blot of EPHB4 in AGS cells upon overexpression of YBX1 wild-type and mutant under IGF2BP1 knockdown conditions. **(B)** Western blot of EPHB4 in AGS cells upon overexpression of IGF2BP1 wild-type and mutant under YBX1 knockdown conditions. **(C)** RT-qPCR of EPHB4 in AGS cells upon overexpression of YBX1 wild-type and mutant under IGF2BP1 knockdown conditions. **(D)** RT-qPCR of EPHB4 in AGS cells upon overexpression of IGF2BP1 wild-type and mutant under YBX1 knockdown conditions. **(E)** RIP-qPCR assay showing EPHB4 mRNA bound to IGF2BP1 or YBX1 upon knockdown of YBX1 or IGF2BP1. **(F)** RIPiT assay showing EPHB4 mRNA bound to IGF2BP1 or YBX1 followed by re-immunoprecipitation with other antibodies. **(G)** m^5^C-MeRIP-qPCR or m^6^A-MeRIP-qPCR confirming m^5^C or m^6^A modification on EPHB4 mRNA following IGF2BP1 or YBX1 knockdown. **(H)** RIP-qPCR assessing IGF2BP1 binding to C100417357A and A100417344C. **(I)** RIP-qPCR assessing YBX1 binding to C100417357A and A100417344C. **(J)** m^6^A-MeRIP-qPCR of EPHB4-CDS and C100417357A to evaluate YBX1 knockdown effect on m^6^A levels. **(K)** m^5^C-MeRIP-qPCR of EPHB4-CDS and A100417344C to evaluate IGF2BP1 knockdown effect on m^5^C levels. *p < 0.05.

Because YBX1 and IGF2BP1 both acted on EPHB4 mRNA, we next asked whether they cooperate functionally. Reciprocal rescue experiments showed that the reduction in EPHB4 expression caused by IGF2BP1 knockdown could be rescued by wild-type YBX1, but not by YBX1 W65A mutant; conversely, wild-type IGF2BP1, but not its mutant form, restored EPHB4 expression after YBX1 depletion (Figures 5A–D). These findings indicate that the two proteins act in a coordinated manner rather than through fully independent pathways.

RIP assays further showed that YBX1 knockdown reduced the binding of EPHB4 mRNA to IGF2BP1, whereas IGF2BP1 knockdown reduced the binding of EPHB4 mRNA to YBX1 (Figure 5E). RIPiT analyses confirmed that YBX1 and IGF2BP1 could be sequentially re-enriched on the same EPHB4 transcript population (Figure 5F), supporting co-occupancy of *EPHB4* mRNA by both factors. Consistent with this interpretation, YBX1 depletion reduced the abundance of m6A-enriched *EPHB4* RNA, whereas IGF2BP1 depletion reduced the abundance of m^5^C-enriched *EPHB4* RNA (Figure 5G). Inspection of the mapped sites revealed a functionally important pair of neighboring residues, C100417357 and A100417344, located within 25 nucleotides of each other. Mutation of either site weakened the association of EPHB4 mRNA with the corresponding reader protein and disrupted the reciprocal influence of YBX1 and IGF2BP1 on m^6^A- and m^5^C-enriched EPHB4 transcripts (Figures 5H–K). These results indicate that YBX1 and IGF2BP1 cooperate on closely positioned RNA elements to maintain EPHB4 expression.

### 3.6 YBX1 physically interacts with IGF2BP1

**FIGURE 6:**
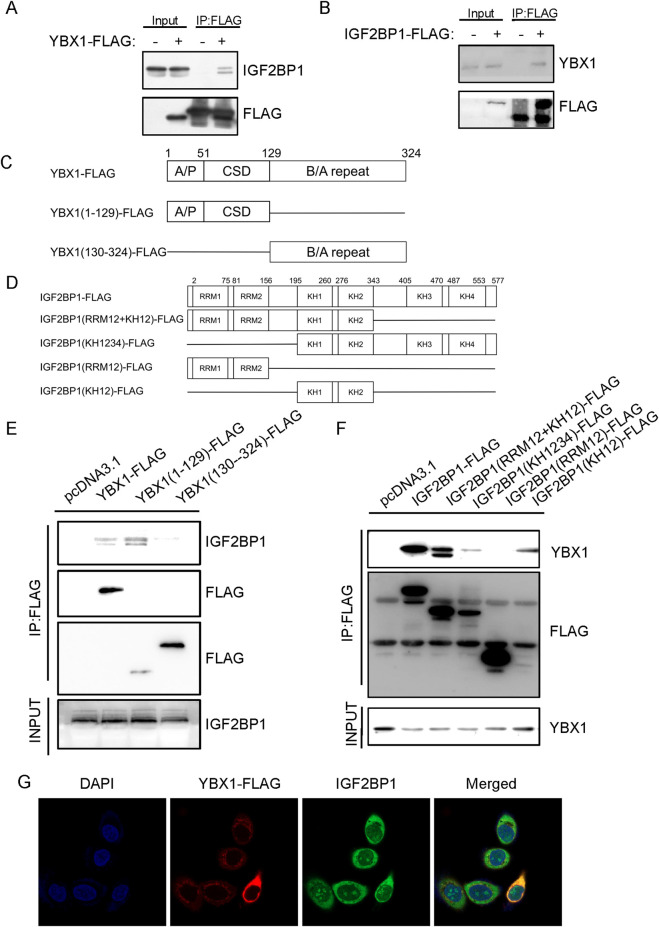
Interaction Between YBX1 and IGF2BP1 **(A)** Western blot detecting the interaction between YBX1-FLAG and endogenous IGF2BP1. **(B)** Western blot detecting the interaction between IGF2BP1-FLAG and endogenous YBX1. **(C)** Schematic diagram of YBX1 deletion mutants. **(D)** Schematic diagram of IGF2BP1 deletion mutants. **(E)** The interaction between YBX1 deletion mutant and endogenous IGF2BP1. **(F)** The interaction between IGF2BP1 deletion mutant and endogenous YBX1. **(G)** Confocal microscopy showing co-localization of YBX1 with IGF2BP1.

To define the basis of YBX1-IGF2BP1 cooperation, we performed co-immunoprecipitation assays. Overexpressed YBX1-FLAG pulled down endogenous IGF2BP1, and IGF2BP1-FLAG reciprocally precipitated endogenous YBX1 (Figures 6A,B), demonstrating a physical interaction between the two proteins. We next mapped the interacting regions using deletion mutants (Figures 6C,D). Co-IP analysis indicated that the N-terminal region of YBX1 (amino acids 1–129) and the KH12-containing region of IGF2BP1 (amino acids 195–343) were required for this interaction (Figures 6E,F). Confocal microscopy further showed that YBX1 and IGF2BP1 predominantly colocalized in the cytoplasm of AGS cells (Figure 6G). Taken together, these data support a model in which physical interaction between YBX1 and IGF2BP1 facilitates coordinated recognition of m^5^C- and m^6^A-associated EPHB4 transcripts, thereby sustaining EPHB4 expression in gastric cancer cells.

## 4 Discussion

Lymphatic metastasis is the dominant route of dissemination in gastric cancer and one of the strongest determinants of staging, recurrence, and survival. In the present study, we identify EPHB4 as a clinically relevant and functionally important driver of gastric cancer lymphatic metastasis. We further show that EPHB4 expression is maintained by a cooperative post-transcriptional program involving NSUN2-dependent m^5^C deposition, YBX1-mediated recognition of m^5^C-marked RNA, and IGF2BP1-associated recognition of m^6^A-modified transcripts. These findings place EPHB4 at the intersection of lymphatic signaling and epitranscriptomic regulation.

EPHB4 has well-established roles in vascular patterning, lymphatic valve development, and endothelial biology (Zhang et al., 2015; Wang, Chen, and Anderson, 1998; He et al., 2005; Martin-Almedina et al., 2016). In cancer, increased EPHB4 expression has been linked to invasion, angiogenesis, and metastasis in multiple tumor types (Alam et al., 2009; Yi et al., 2021; Liu et al., 2018; Ullah et al., 2024; Chen, Zhang, and Zhang, 2019). Our data extend these observations in gastric cancer by showing that EPHB4 is enriched in lymph node-positive tumors, promotes metastasis to regional draining lymph nodes *in vivo*, and enhances migration and invasion *in vitro*. The cell-surface crosslinking experiments further suggest that tumor-cell EPHB4 may directly engage molecules on lymphatic endothelial cells, consistent with prior evidence that EPHB4-ephrin-B2 signaling contributes to lymphatic biology (Zhang et al., 2015; Oliver et al., 2020; Chen et al., 2023). Together, these observations support a model in which EPHB4 promotes lymphatic dissemination both by strengthening tumor-intrinsic motility and by facilitating tumor-endothelial interactions.

A major advance of this study is the identification of a dual RNA-modification mechanism that converges on EPHB4 mRNA. NSUN2 has previously been shown to enhance the stability of oncogenic transcripts through m^5^C deposition (Mei et al., 2020), whereas YBX1 and IGF2BP family proteins act as modification-sensitive RNA effectors that stabilize selected target RNAs (Qin et al., 2024; Luo and Lin, 2022). Here, we show that NSUN2 is required for m^5^C deposition on *EPHB4* mRNA, that YBX1 recognizes the NSUN2-responsive transcript, and that IGF2BP1 preferentially associates with m6A-enriched *EPHB4* RNA. Mapping analyses further identify discrete functional m^5^C- and m^6^A-associated sites, indicating that EPHB4 is not simply a generic target of these regulators but a transcript controlled through specific cis-elements. This mechanism provides a direct explanation for how multiple RNA modification pathways can converge to amplify a metastasis-promoting receptor in gastric cancer.

Our data also support a more integrated model of epitranscriptomic crosstalk (Song et al., 2021; Huang, Meng, and Chen, 2024; Zhang et al., 2025b). YBX1 and IGF2BP1 not only rescued each other’s loss in reciprocal complementation assays, but also co-occupied *EPHB4* mRNA, physically interacted with one another, and reciprocally influenced the abundance of m^5^C- and m^6^A-enriched EPHB4 transcripts. The proximity of the critical C100417357 and A100417344 residues suggests that closely spaced modification-associated elements may create a local RNA environment that favors cooperative assembly of stabilizing ribonucleoprotein complexes. This type of cooperation may help explain how modest effects exerted by individual RNA-binding proteins can be integrated into a robust increase in transcript stability and metastatic capacity.

From a translational perspective, the NSUN2-YBX1-IGF2BP1-EPHB4 axis offers several potential intervention points. EPHB4 is a membrane receptor with established druggability and has already been explored as a therapeutic target in cancer (Ullah et al., 2024; Chen, Zhang, and Zhang, 2019; Sadeghi et al., 2023; VanderWeele et al., 2022). Targeting the upstream RNA regulatory machinery could offer an additional strategy to suppress EPHB4 expression before receptor signaling is engaged. At the same time, our study has limitations. The clinical cohort was relatively modest in size, and both the *in vivo* experiments and the clinical validation cohort were restricted to male subjects; thus, the extent to which these findings apply to female patients warrants further investigation. The clinical cohort is relatively modest, and the mechanistic conclusions would be further strengthened by orthogonal site-validation approaches and by therapeutic perturbation studies *in vivo*. Nonetheless, the present work defines a coherent mechanistic framework in which m^5^C- and m^6^A-related regulators cooperate to stabilize EPHB4 mRNA and thereby promote gastric cancer lymphatic metastasis. These findings expand the biological understanding of lymphatic dissemination and support the development of epitranscriptomic strategies for patients with lymph node-positive gastric cancer.

## 5 Conclusion

This study defines a cooperative epitranscriptomic mechanism that drives lymphatic metastasis in gastric cancer. Through integrated clinical, functional, and mechanistic analyses, we identified EPHB4 as a key metastasis-promoting factor and demonstrated that its expression is jointly sustained by NSUN2/YBX1-mediated m^5^C regulation and IGF2BP1-associated m^6^A recognition. We further showed that YBX1 and IGF2BP1 cooperate physically and functionally on EPHB4 transcripts, highlighting a previously unrecognized interaction between m^5^C- and m^6^A-related regulatory pathways. Collectively, these findings deepen the mechanistic understanding of gastric cancer lymphatic dissemination and support the NSUN2-YBX1-IGF2BP1-EPHB4 axis as a potential biomarker and therapeutic target in lymph node-positive gastric cancer.

## Data Availability

Publicly available datasets were analyzed in this study. This data can be found here: https://ngdc.cncb.ac.cn/gsa-human/browse/HRA014173.
