# Supplementary material for: m5C and m6A cooperatively stabilize EPHB4 to drive lymphatic metastasis in gastric cancer
Source: Front Genet. 2026 May 20;17:1845032. doi: 10.3389/fgene.2026.1845032 (PMC13218831; doi:10.3389/fgene.2026.1845032)
Supplement: Supplementary file 1 [file Table1.docx]

**SUPPLEMENTARY TABLES**

**Table S1. The sequences for shRNAs or siRNA**

| **shRNA** | **Sequence (5’-3’)** |  |
| --- | --- | --- |
| ShEPHB4-1 | accggtCACGAGCTCCCTGGGAGGAAActcgagTTTCCTCCCAGGGAGCTCGTGtttttttgaattc | |
| shEPHB4-2 | accggtCCCAGCCAATAGCCACTCTAActcgagTTAGAGTGGCTATTGGCTGGGttttttgaattc | |
| shNC | accggtTTCTCCGAACGTGTCACGTTTCAAGAGAACGTGACACGTTCGGAGAAttttttgaattc | |
| siEPHB4-1 | CACGAGCUCCCUGGGAGGAAATT | |
| siEPHB4-2 | CCCAGCCAAUAGCCACUCUAATT | |

**Table S2. The primer sequences for RT-qPCR**

| **Gene (Homo)** |  | **Sequence (5’-3’)** |
| --- | --- | --- |
| EPHB4 | Sense | TCCTGCAAGGAGACCTTCAC |
|  | Anti-sense | GTGCAGGGATAGCAGGGCCAT |
| NSUN2 | Sense | ATCTTGAGAAAATCGCCACA |
|  | Anti-sense | ATCATTCGCAATAACAAATCCCT |
| YBX1 | Sense | CGGAGGCAGCAAATGTTACA |
|  | Anti-sense | GACCCCTACGACGTGGATAG |
| GAPDH | Sense | AGCCACATCGCTCAGACAC |
|  | Anti-sense | GCCCAATACGACCAAATCC |
| IGF2BP1 | Sense | TTACTGGGGCTGCTCCCTAT |
|  | Anti-sense | TTCGGGTGGTGCAATCTTGA |
| EPHB4-m^5^C-5’UTR-1 (100424931,100424933) | Sense | AAGACGTGGCTGGAGTTGG |
|  | Anti-sense | GAGCGGCCGGCTCAG |
| EPHB4-m^5^C-CDS-1  (100404177) | Sense | CCTCCGGGGCAGTCCATC |
|  | Anti-sense | GAAAAAGCAGAGGCAGGTGG |
| EPHB4-m^5^C-CDS-2  (100417357) | Sense | AGGCGGGAAACCACGCTC |
|  | Anti-sense | GTGGAGCCCACTGTCTCCAAG |
| EPHB4-m^5^C-CDS-3  (100420273) | Sense | CGCAGCGTCTTGACATTCAC |
|  | Anti-sense | GAACGGGAACCAAGGAGGTC |
| EPHB4-m^5^C-3’UTR-1  (100400612, 100400613, 100400652) | Sense | AGGGCCCAGTGACAAAATCA |
|  | Anti-sense | GGGGAGGGGCATTTACAAGG |
| EPHB4-m^5^C-3’UTR-2  (100400592, 100400589) | Sense | CTTGTAAATGCCCCTCCCCC |
|  | Anti-sense | ACAGGTTCCCTCCAACACAAA |
| EPHB4-m^5^C-3’UTR-3  (100400394, 100400393, 100400387) | Sense | TCACTATGGCCTCCTTTGCC |
|  | Anti-sense | GGGGATGTGGGATGACCAAG |
| pmirGLO-EPHB4-3’UTR | Sense | TAGTTGGTGGTGGAACCCA |
|  | Anti-sense | TCCTTTCGGGCTTTGTTAG |
| pmirGLO-EPHB4-CDS | Sense | GGCAAGATCGCCGTGTAATTC |
|  | Anti-sense | :CACCTGAGGGAATGTCACCC |
| EPHB4-CDS-m^6^A-1  (100421545) | Sense | AGAGACCCTGCTGAACACA |
|  | Anti-sense | CGTCACACACTTCGTAGGTG |
| EPHB4-CDS-m^6^A-2  (100420200) | Sense | CCACCGGGAAGGTGAATGTC |
|  | Anti-sense | GAAGGCCAGGTAGAAGCCAG |
| EPHB4-CDS-m^6^A-3  (100420100, 100420088) | Sense | TGCTATCCCTGCACCTCTTC |
|  | Anti-sense | CACAGTCTCCGGGAATCGAG |
| EPHB4-CDS-m^6^A-4  (100417843) | Sense | CAAGCCCCTGTCAGGAGAAG |
|  | Anti-sense | ACGGCTGATCCAATGGTGTT |
| EPHB4-CDS-m^6^A-5  (100417344, 100417322) | Sense | GAGGCTCCTGTGCGCC |
|  | Anti-sense | GTGAAGTCAGGACGTAGCCC |
| EPHB4-CDS-m^6^A-6  (100405087) | Sense | ACTCCTTCCTGCGGCTAAA |
|  | Anti-sense | CAGACGAGGTTGCTGTTGA |
| EPHB4-CDS-m^6^A-7  (100403222, 100403177) | Sense | ATGCTGGACTGTTGGCAGAA |
|  | Anti-sense | CCCACAGAGCCAAAAGCTGA |
| EPHB4-CDS-m^6^A-8  (100401121, 100401109, 100401083, 100401057, 100401023) | Sense | GTCCAGCACATGAAGTCCCA |
|  | Anti-sense | GGCTCAAAGTGCAATCCAGC |
| EPHB4-3’UTR-m^6^A-1  (100400944, 100400883, 100400847) | Sense | GCTGGATTGCACTTTGAGCC |
|  | Anti-sense | TGGGAGATGTTGGGCACTTC |
| EPHB4-3’UTR-m^6^A-2  (100400677) | Sense | TTCCCGCAGACCAAAGAGAG |
|  | Anti-sense | GGGGGAGGGGCATTTACAAG |
| EPHB4-3’UTR-m^6^A-3  (100400400) | Sense | TCACTATGGCCTCCTTTGCC |
|  | Anti-sense | GGGGATGTGGGATGACCAAG |

**Supplementary Table 3**

|  | **baseMean** | **log2FoldChange** | **p-value** | **Gene ID** |
| --- | --- | --- | --- | --- |
| 1 | 1051.713 | 0.116677 | 2.76E-06 | UPK1B |
| 2 | 821.4798 | 0.41276 | 0.005779 | CCR7 |
| 3 | 1672.462 | 2.39515 | 0.010001 | KIF11 |
| 4 | 1204.001 | 0.324736 | 0.023797 | CCL21 |
| 5 | 468.995 | 0.385947 | 0.029364 | GJC2 |
| 6 | 401.6074 | 0.284474 | 0.048307 | CXCR5 |
| 7 | 29807.26 | 1.781267 | 0.050523 | EPHB4 |
| 8 | 11441.68 | 1.361172 | 0.070269 | DDX3X |
| 9 | 10618.86 | 1.518019 | 0.078196 | HNRNPA2B1 |
| 10 | 256.4201 | 0.370553 | 0.082138 | LYVE1 |
| 11 | 1555.886 | 2.060327 | 0.084898 | FOXC1 |
| 12 | 44.89237 | 0.500643 | 0.085479 | CCBE1 |
| 13 | 2183.969 | 1.474987 | 0.086956 | QKI |
| 14 | 11103.48 | 1.426843 | 0.087943 | SRSF1 |
| 15 | 1710.358 | 0.60607 | 0.116342 | ADM |
| 16 | 45.77981 | 1.990506 | 0.124164 | CCR8 |
| 17 | 3822.381 | 1.378648 | 0.134935 | ITGA9 |
| 18 | 5738.073 | 1.54476 | 0.135621 | HMGB1 |
| 19 | 33167.35 | 1.340699 | 0.151552 | PIEZO1 |
| 20 | 1970.32 | 0.611961 | 0.162081 | ADAM33 |
| 21 | 8369.274 | 1.389272 | 0.163733 | ESRP1 |
| 22 | 947.9231 | 1.655538 | 0.191842 | PROX1 |
| 23 | 697.3575 | 0.747914 | 0.20409 | FBXL7 |
| 24 | 279.0162 | 0.699883 | 0.226166 | FABP5 |
| 25 | 5490.833 | 1.329957 | 0.244155 | PTPN14 |
| 26 | 3863.038 | 1.281915 | 0.254427 | DNMT3A |
| 27 | 32068.39 | 0.664154 | 0.272348 | GATA6 |
| 28 | 5762.438 | 0.741966 | 0.27398 | CXCR4 |
| 29 | 8118.644 | 1.35521 | 0.295286 | LAMC2 |
| 30 | 3882.697 | 1.340854 | 0.309685 | TIMP1 |
| 31 | 42.23184 | 2.079786 | 0.333345 | TWIST1 |
| 32 | 6.608716 | 3.808142 | 0.340612 | VEGFD |
| 33 | 1506.357 | 0.747497 | 0.349383 | FLT4 |
| 34 | 171.0046 | 0.673867 | 0.369226 | VEGFC |
| 35 | 29864.55 | 1.208498 | 0.369222 | ANXA2 |
| 36 | 321.2256 | 0.690481 | 0.388268 | CXCR3 |
| 37 | 73239.93 | 1.345498 | 0.40337 | ALDOA |
| 38 | 8559.548 | 1.194845 | 0.444808 | ANXA5 |
| 39 | 410.7807 | 0.736018 | 0.454335 | IL7 |
| 40 | 8622.142 | 1.175712 | 0.477592 | IMPDH2 |
| 41 | 235.3555 | 0.630137 | 0.48046 | CXCR2 |
| 42 | 1211.82 | 0.769636 | 0.510707 | CXCL12 |
| 43 | 1490.664 | 1.282926 | 0.516716 | GATA2 |
| 44 | 602.5375 | 1.166356 | 0.587213 | IGF1 |
| 45 | 5359.452 | 0.830659 | 0.587688 | GDF15 |
| 46 | 11309.52 | 0.86877 | 0.603248 | ESRP2 |
| 47 | 9839.464 | 0.896278 | 0.6158 | IGFBP3 |
| 48 | 32077.22 | 1.087883 | 0.617202 | PTBP1 |
| 49 | 4462.489 | 1.120276 | 0.648354 | RBFOX2 |
| 50 | 2101.16 | 1.136149 | 0.652722 | SOX18 |
| 51 | 66.13439 | 1.359957 | 0.656479 | ADAMTS3 |
| 52 | 277.6892 | 1.187548 | 0.680459 | SNAI1 |
| 53 | 9051.509 | 0.877985 | 0.696339 | TIMP3 |
| 54 | 2139.505 | 0.914065 | 0.706098 | APC |
| 55 | 4153.376 | 1.092873 | 0.747647 | FAT4 |
| 56 | 2332.682 | 1.101634 | 0.774168 | ANXA1 |
| 57 | 4407.841 | 0.943358 | 0.788837 | IMP3 |
| 58 | 1178.733 | 1.114092 | 0.789645 | PDPN |
| 59 | 95.88932 | 1.18193 | 0.790132 | FOXC2 |
| 60 | 677.0314 | 0.922721 | 0.807518 | SPHK1 |
| 61 | 2092.708 | 0.94465 | 0.810124 | PDGFB |
| 62 | 534.8954 | 0.909497 | 0.813262 | ANGPT2 |
| 63 | 1277.106 | 1.059137 | 0.831359 | ZEB1 |
| 64 | 11051.74 | 0.963032 | 0.83329 | STAT3 |
| 65 | 53581.82 | 1.059274 | 0.833758 | FASN |
| 66 | 1737.489 | 0.917594 | 0.836159 | IGF2 |
| 67 | 31645.31 | 1.046195 | 0.855416 | EZR |
| 68 | 285.2033 | 1.085452 | 0.890599 | HGF |
| 69 | 9092.573 | 1.028153 | 0.90123 | ADAM9 |
| 70 | 377.4155 | 0.954722 | 0.908538 | ADAM12 |
| 71 | 23329.44 | 1.018966 | 0.922599 | ADAM15 |
| 72 | 775.8398 | 1.034492 | 0.927421 | CALCRL |
| 73 | 12592.42 | 1.016708 | 0.94121 | TIMP2 |
| 74 | 292.6922 | 0.969835 | 0.958853 | SNAI2 |
| 75 | 1898.071 | 1.03021 | 0.962898 | MMP1 |
| 76 | 45288.14 | 1.005998 | 0.972417 | EIF4G1 |
| 77 | 0 | NA | NA | SOX3 |
| 78 | 3603.106 | 4.529185 | NA | SMARCE1 |
| 79 | 0 | NA | NA | CCL1 |

**Table S4. The m^5^C sites of EPHB4**

| **m^5^C Site** | Gene Region | **m^5^C-Atlas** | **Sequence*** |
| --- | --- | --- | --- |
| 100400387 | 3’UTR | + | CCT TGG TCA TCC CAC |
| 100400393 | 3’UTR | + | ACA GTG CCT TGG TCA |
| 100400394 | 3’UTR | + | AAC AGT GCC TTG GTC |
| 100400589 | 3’UTR | + | CCC CCA GCT GCT GCC |
| 100400592 | 3’UTR | + | CCT CCC CCA GCT GCT |
| 100400612 | 3’UTR | + | TTT TTT CCC TTG TAA |
| 100400613 | 3’UTR | + | TTT TTT TCC CTT GTA |
| 100404177 | EXON | + | AGA TTC CCA TCC GAT |
| 100417357 | EXON | + | CGC CCT GCG GGG GAG |
| 100420273 | 5’UTR | + | GTG GCC GCG GAG CAT |
| 100424931 | 5’UTR | + | CGA GGC CCC GGA GGG |
| 100424933 | 5’UTR | + | CCC GAG GCC CCG GAG |

*The m^5^C sites were underlined.

**Table S5. The m^6^A sites of EPHB4**

| **m^6^A Site** | Gene Region | **m^6^A-Atlas** | **Sequence*** |
| --- | --- | --- | --- |
| 100400400 | 3’UTR | + | TTC CAG AAC AGT GCC |
| 100400677 | 3’UTR | + | CCC AGT GAC AAA ATC |
| 100400847 | 3’UTR | + | CCT CAG GAC TGG GTG |
| 100400883 | 3’UTR | + | TCG GGG AAC TCC AGA |
| 100400944 | 3’UTR | + | TGG AGA GAC AGG ATT |
| 100401023 | 3’UTR | + | AGT GGG GAC TCA CAG |
| 100401057 | 3’UTR | + | CCC AGG GAC ACC GCC |
| 100401083 | EXON | + | AGT ACT GAC CTG CAG |
| 100401109 | EXON | + | GGG TGG GAC AGG AGG |
| 100401121 | EXON | + | GCC GGG AAC CCC GGG |
| 100403177 | EXON | + | GCC CTG GAC AAG ATG |
| 100403222 | EXON | + | CAG AAA GAC CGG AAT |
| 100405087 | EXON | + | GCT CGC AAC ATC CTA |
| 100417322 | EXON | + | CCC CGG GAC CTG GTG |
| 100417344 | EXON | + | AGA CCT GAC TTT TGA |
| 100417843 | EXON | + | CAC TCT AAC ACC ATT |
| 100420088 | EXON | + | GAA CCT GAC TCG ATT |
| 100420100 | EXON | + | CCA GCT GAC TGT GAA |
| 100420200 | EXON | + | GTC TGG GAC CGC TCA |
| 100421545 | EXON | + | GGG AGG AAC TGA GCG |

*The m^6^A sites were underlined.
